# The Rapid Initial Community Builder (RICB) V1.1 for LANDIS-II

**DOI:** 10.1016/j.mex.2025.103765

**Published:** 2025-12-13

**Authors:** Steven A Flanagan, Zachary J. Robbins, Mac A. Callaham, J. Kevin Hiers, Brian R. Miranda, Joseph J. O’Brien, Robert M. Scheller, E. Louise Loudermilk

**Affiliations:** a Disturbance and Prescribed Fire Laboratory, Southern Research Station, U.S. Forest Service, Athens, GA, USA; b Earth & Environmental Sciences Division, Los Alamos National Laboratory, Los Alamos, NM, USA; c Natural Resources Institute, Texas A&M University, Washington, DC 20006, USA; d Eastern Planning Service Group, U.S. Forest Service, La Crosse, WI, USA; e Department of Forestry and Environmental Research, North Carolina State University, Raleigh, NC, USA

**Keywords:** TreeMap, Initial communities, FIA, Forest landscape model, LANDIS-II, Ecosystem process model

## Abstract

Forest succession, or ecosystem process models, can aid in many land management decisions as they inherently incorporate ecological processes to predict how disturbances impact the resilience of the forest and its potential future structure. The landscape class of ecosystem process models are often ideal for management goals as they simulate individual tree species interactions through time at the landscape scale and offer a robust assortment of disturbance extensions. However, broad implementation and adoption are often limited as users must rebuild the landscape models for every new site of interest. Creation of the initial forest communities that all model simulations depend on, can be particularly time consuming. To address this issue, we created the Rapid Initial Community Builder (RICB) that:

• Starts with high resolution CONtinental United States (CONUS) forest coverage and type data.
• Makes modifications to the data for use in the landscape class ecosystem process model LANDIS-II.
• Packages everything in an executable file that eliminates coding language or version barriers.

Thus, greatly reducing the initialization time often associated with the intensive data assimilation techniques commonly used to generate initial communities.

## Graphical abstract

**Figure:**
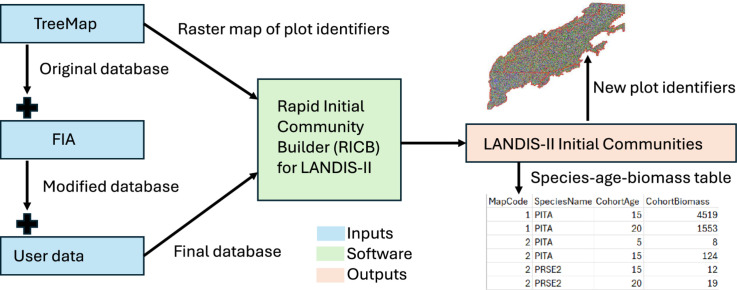

**Specifications table**

**Table:** 

**Subject area**	Environmental Science
**More specific subject area**	Landscape ecosystem process modeling
**Name of your method**	Rapid Initial Community Builder (RICB)
**Name and reference of original method**	Not Applicable
**Resource availability**	RICB and the code generated to build it is available on Zenodo: 10.5281/zenodo.15685152

## Background

The landscape class of forest succession models, or forested landscape models, are ideal for informing land managers on how the structure of their forests could potentially change under different disturbance regimes. The LANdscape Disturbance and Succession v.2 (LANDIS-II) [1] model is of this class and has been frequently used to address various research and land management questions [[2], [3], [4]]. This is because LANDIS-II simulates individual tree species interactions through time (monthly to decadal time steps that can run for centuries) over medium to large sized areas (hundreds to millions of acres) and offers a robust assortment of disturbance extensions related to fire, disease outbreaks, weather patterns, timber harvest, hurricanes, and others [[5], [6], [7], [8], [9], [10], [11]]. However, broad implementation and adoption has been limited as modelers must rebuild the landscape (soil and weather properties; initial tree communities – type, age, amount) for every new geographic site of interest, which typically takes 9 months to a year [12].

The generation of the initial tree communities input file of species-age-biomass cohorts required to run the model and on which all outputs depend accounts for most of this long initialization time. Standard methods of constructing these inputs include collecting field data directly or having an organization provide it indirectly [13] at a resolution necessary to run the model, imputing coarse scale forest inventory to a fine scale resolution using landscape data (topographic, biophysical, and disturbance) directly [14,15] or adjusting a high-resolution imputation not specifically constructed for LANDIS-II [16] to be compatible. Regardless of the method chosen, there have only been limited efforts [17] to create a Graphical User Interface (GUI) where future users do not need to worry about what language and version the scripts used to generate the inputs were written in to generate these files. However, these are often condensed to the minimal details required to construct initial communities and thus still require additional user input. Here, we introduce the Rapid Initial Community Builder (RICB) for LANDIS-II. It is a GUI that allows users to import several standard Geographical Information Science (GIS) data types (shapefiles, tiff, and kml) and automatically builds the species-age-biomass cohorts and spatial distribution map based on a user’s specifications. The final application thus supports the rapid creation of LANDIS-II initial communities without requiring the extensive data assimilation techniques commonly used in these projects and removes any coding language or version barriers.

## Method details

### TreeMap database modification for LANDIS-II initial communities: FIA

TreeMap [18,19] is a gridded CONtinental United States (CONUS) dataset that imputed reference Forest Inventory and Analysis (FIA) data to target LANDFIRE data (location, vegetation, biophysical, disturbance, and topographic properties) using a random forest machine learning algorithm. The final product provides a raster of wall-to-wall coverage of CONUS predictions of what FIA plot best represents any given forested pixel at 30 m x 30 m resolution and an accompanying database file with some key information about each tree in each FIA plot. The database was specifically constructed to provide users with the utility to access and link additional FIA characteristics through the FIA DataMart [20]. While many of the attributes necessary to construct the species-age-biomass cohorts that LANDIS-II requires for its initial communities are provided in the original database files, additional data was required and accessed through this linkage. Therefore, several attributes required for LANDIS-II, or deemed useful, were added to the original database (Table 1) files provided by TreeMap. The only attribute not obtained from the FIA archive file “REF_SPECIES.csv” was “SITE_INDEX”, which is found in the SQLite Database under “FVS_PLOTINIT_PLOT”. The code to perform the expansion of the database is found on the resource availability website. TreeMap has two available versions, referred to as TreeMap 2014 or 2016. The same code is used for both database files, but TreeMap 2016 increased the number of attributes it provided compared to the 2014 version. As such, there is some redundancy in the attributes we added and the 2016 database.

**Table 1:** Additional FIA attributes added to the TreeMap database, their descriptors, and what they were used for to create LANDIS-II initial community inputs.

FIA Attribute	Descriptor	Use
COMMON_NAME	Common name of tree species	Reference
GENUS	Genus	Reference
SPECIES	Species	Reference
SPECIES_SYMBOL	USDA PLANTS Database code	LANDIS-II Cohort code
MAJOR_SPGRPCD	Major species group code	Reference
W_SPGRPCD	Western species group code	Reference
E_SPGRPCD	Eastern species group code	Age calculation
SITE_INDEX	Site index	Age calculation
JENKINS_SPGRPCD	Jenkins biomass species group code	Biomass calculation
JENKINS_TOTAL_B1	Jenkins coefficient 1	Biomass calculation
JENKINS_TOTAL_B1	Jenkins coefficient 2	Biomass calculation

### TreeMap database modification for LANDIS-II initial communities: non-FIA

Carmean’s site index equations [21] are commonly used by LANDIS-II researchers to calculate ages from FIA data [9,[22], [23], [24]]. However, they are species specific which are not feasible when accounting for all species in the TreeMap of FIA databases. Therefore, to minimize the total area and hence number of species that required parameterization and because Carmean primarily focused on eastern species, we focused here on the eastern U.S. as defined by the United States Forest Service (USFS). Region 8 (AL, AR, FL, GA, KY, LA, MS, NC, OK, SC, TN, TX, VA) and 9 (CT, DC, DE, IA, IL, IN, MA, MD, ME, MI, MN, MO, NH, NJ, NY, OH, PA, RI, VT, WI, WV) which encompasses 34 states on the U.S. eastern seaboard were chosen as our test area. To further reduce the number of species required for parameterization a representative species was chosen for each Eastern Species GRouP CoDe (E_SPGRPCD) as determined by what species with that code had the highest number of individuals in each region as calculated by TreeMap 2014 (Table 2). Thus, mapping all species present from each E_SPGRPCD into a single representative species of that code. Each region was run separately to check for potential latitudinal gradients in the change of dominant species by species group code, but as only 6 of the 30 species changed when moving from region 8 to 9, only the region 8 results are provided. The differences are provided in the Supplementary Materials (Table S1). If a species had multiple possible site index curves the coefficients that fit the greatest number of trees were chosen (Table S2). The coefficients related to each E_SPGRCD to calculate age were then added to the adjusted TreeMap database.

**Table 2:** The dominant species by total number of trees per FIA eastern species group code in USFS Region 8 that were used to calculate age.

Region 8 Representative Species for each FIA E_SPGRPCD					
Code	Common Name	Code	Common Name	Code	Common Name
1	longleaf pine	25	white oak	35	blackgum
2	shortleaf pine	26	northern red oak	36	green ash
3	virginia pine	27	chestnut oak	37	eastern cottonwood
4	eastern white pine	28	water oak	38	American basswood
5	jack pine	29	mockernut hickory	39	yellow-poplar
6	red spruce	30	yellow birch	40	black walnut
7	eastern hemlock	31	sugar maple	41	black cherry
8	pondcypress	32	red maple	42	black locust
9	eastern redcedar	33	American beech	43	sourwood
23	common pinyon	34	sweetgum	48	honey mesquite

### Biomass and age calculations

The combined database was then used to calculate the age and biomass of every alive tree. Biomass was calculated by combining the Jenkins coefficients obtained in the additional FIA data (Table 1) with the originally provided diameter and using Jenkin’s Equation [25]:

$$bm=Exp\left(B_{1}+B_{2}\ln\left(DIA*2.54\right)\right)$$

Where:

• bm is the total aboveground biomass in kilograms dry weight
• B_1_ and B_2_ are the JENKINS_TOTAL_B1 and B2 coefficients from Table 1
• DIA is the diameter at breast height in inches from the original database and 2.54 the scalar to convert to centimeters
• Exp is the exponential function
• ln is log base *e* (2.718282)

And age was calculated from Carmean’s [21] site index equation:

$$age=\frac{1}{b3}*ln\left(1-{\left(\frac{HT}{b1*SI^{b2}}\right)}^{{\frac{SI}{b4}}^{-b5}}\right)$$

Where:

• age is tree age in years
• HT is the height in feet of a given tree from the original database
• SI is the Site_Index from Table 1
• b1, b2, b3, b4, and b5 are the coefficients from Table S2

For computational efficiency, every FIA plot found in the database was then saved as a separate file with these calculated values and some reference information (Table 3) as a “landis_tile” for RICB to reference. The result being every value on the imputed TreeMap raster now has a corresponding “landis_tile” that contains all the information needed for RICB to construct the initial communities. As biomass was calculated based on each individual species FIA assigned JENKINS_SPGRPCD (Table 1) no modifications are needed for this value if a user were to want to use the “landis_tiles” inside of CONUS but outside of USFS Regions 8 and 9. Age was determined by essentially assigning a site index code to each E_SPGRPCD. If a user were comfortable with applying these eastern codes to western species, the “landis_tiles” need no modification for westward expansion. Otherwise, the pre-processing code is provided on the resource website where with modifications to table S2 our work could be repeated to generate western “landis_tiles”.

**Table 3:** An example of a “landis_tile” created from the augmented TreeMap database. Each tile, or file, is a number that corresponds to its FIA plot identifier where age and biomass for each tree (a row is a tree) that RICB will use to construct LANDIS-II species-age-biomass cohorts. It also contains multiple reference columns a user might find useful from Table 1, and a blank “MapCode” column that is used by RICB.

MapCode	SPECIES_SYMBOL	COMMON_NAME	E_SPGRPCD	age	biomass
	QUNI	water oak	28	14.97	26.99
	QUNI	water oak	28	8.21	3.89
	QUNI	water oak	28	15.7	22.94
	QUNI	water oak	28	14.97	13.05
	PITA	loblolly pine	2	14.78	76.79
	QULA3	laurel oak	28	17.60	140.23
	QUNI	water oak	28	8.94	2.67

### RICB

To prevent any coding language and version incompatibilities PyInstaller (v 6.1.1 [26]) was used to bundle our Python code and all its dependences into a single package. Meaning the user does not need to install any additional software or modules. Rather, an executable file with pop-up windows guides them. However, RICB currently only runs on Windows operating systems as PyInstaller is not a cross-compiler. Additionally, as the native 30 m x 30 m resolution of TreeMap may be computationally intensive for large domains, a 300 m x 300 m resolution map as calculated by mode was also generated.

*Step 1: Selection of Region, Spatial Resolution, TreeMap version, and User GIS file*

**Fig. 1:**
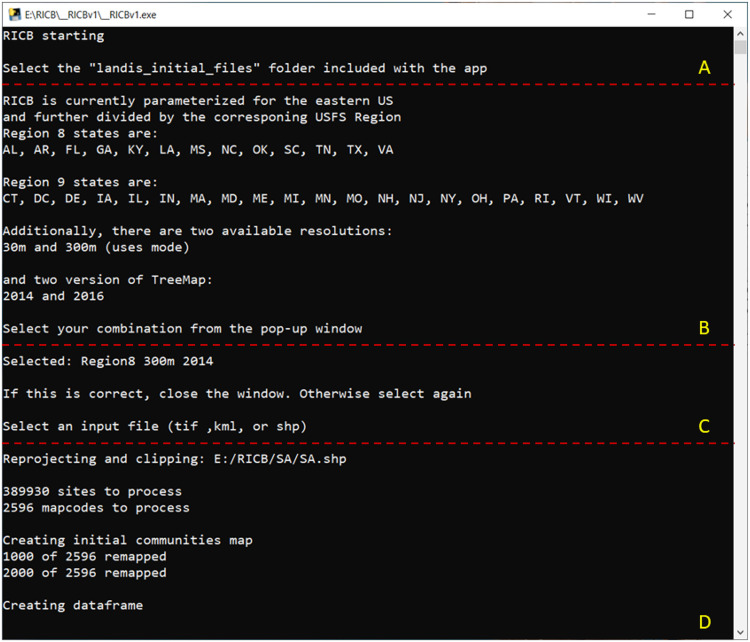
A RICB launched shell (A); that guides a user to select a folder that contains other files needed to run (B); the users desired region, resolution, and TreeMap version combination (C); and the GIS area of interest file where the initial communities build will occur (D).

RICB has two main sub-folders, “__RICBv1” which contains the application/executable file and “landis_initial files” which contains the “landis_tiles” (Table 3) and maps for all possible TreeMap version, resolution, and region combinations a user can select. To start RICB, navigate to and select the executable file (application) in the “__RICBv1” folder (__RICBV1.exe). A command prompt window will launch (Fig. 1A) to guide the user through the process. Multiple pop-up windows will then appear to select the “landis_initial_files” folder (tells RICB where on the computer it should be executing: Fig. 1B), a Region/Resolution/TreeMap version prompt (tells RICB what maps it should be using: Fig. 1C), and a prompt for the user to import various GIS data types (tells RICB what file to use for data extraction: Fig. 1D). After selections are made, RICB reprojects the GIS file and clips the associated map. Then it remaps the values to be sequential and creates a dataframe that is a concatenation of all the landis_tiles (MapCodes in Fig. 1A) that will be used to construct the initial communities. If RICB has previously been run a check to overwrite a “temp_files” directory appears in the prompt to prevent deletion of previously constructed initial communities.

*Step 2: Selection of Species to Include in the Initial Communities Outputs*

**Fig. 2:**
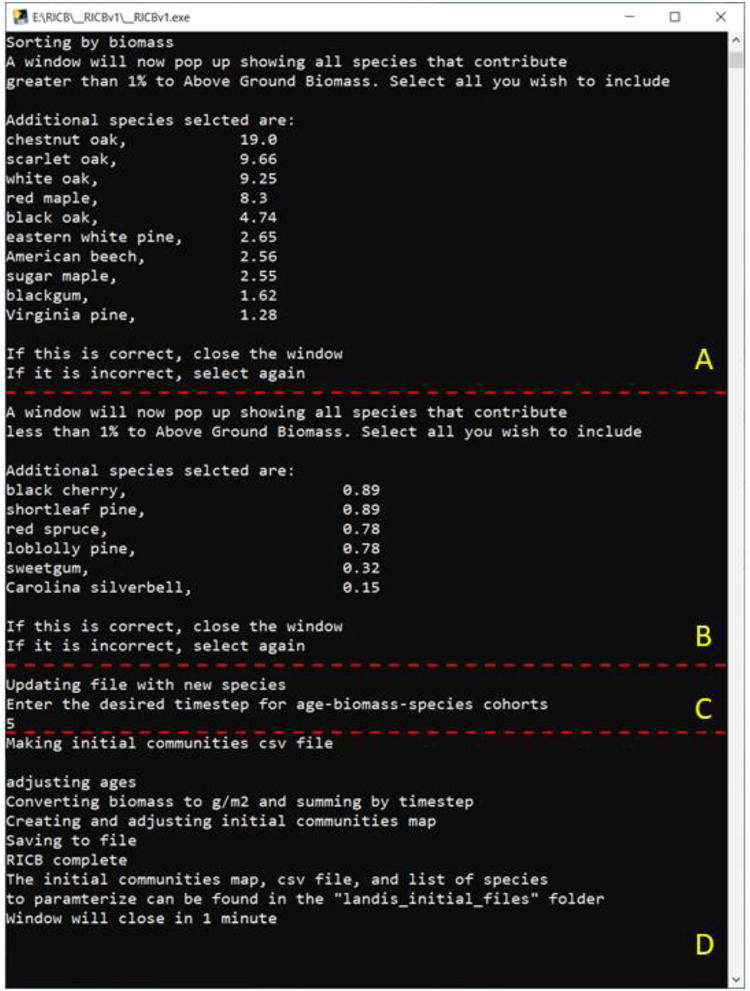
RICB sorts all species by relating that species biomass in the domain to the total biomass in the domain and then generates two pop-up windows (Fig. 3, corresponds to parts A and B) for the user to select which species they desire to include in the final initial communities file. Then the user selects the age interval (C) for the species-age-biomass cohorts, and lastly (D) makes adjustments to the final map and lookup table as needed.

Once the initial communities map and associated dataframe file are built, RICB will then assist the user in the selection of potentially relevant species, ask for the age interval to group cohorts at, and adjust the final map and associated lookup table as needed (Fig. 2). At this point, the RICB dataframe contains ALL species found in the geographic area of interest. Any species listed in this file will require additional parameterization depending on the succession extension(s) the user decides to use. It often will not be feasible, or desirable, to parametrize every species on the landscape. Therefore, RICB first returns a list of all species where the Above Ground Biomass (AGB) is greater than 1 % (Fig. 2B) with respect to total biomass in the domain, which are often the species a user is most interested in The 1 % value was arbitrarily chosen to highlight the species with large AGB values on the landscape. However, as niche species by total AGB are sometimes relevant depending on the research or management question guiding a study, a second list then appears (Fig. 2C) providing the user with the opportunity to select any species, including those <1 %. Users will often need to scroll through this window to view every possible species. RICB verifies the users’ selections and ONLY those selected will appear in the final initial communities file required to run LANDIS-II.

*Step 3: Generating the Initial Communities File and Associated Map.*

**Fig. 3:**
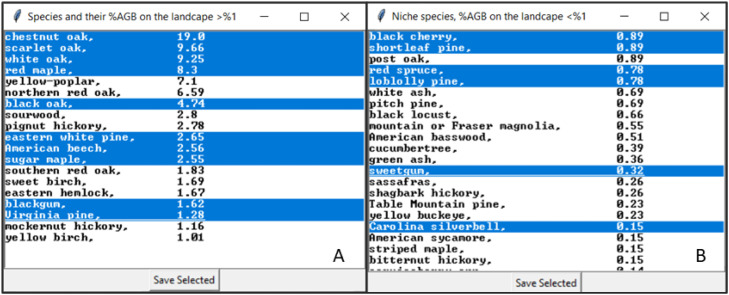
The pop-up windows (Fig. 2A and B) RICB generates where a user selects what species that have greater than 1 % (A) and <1 % (B) of the total biomass on the landscape to use in the final initial communities LANDIS-II lookup table.

**Fig. 4:**
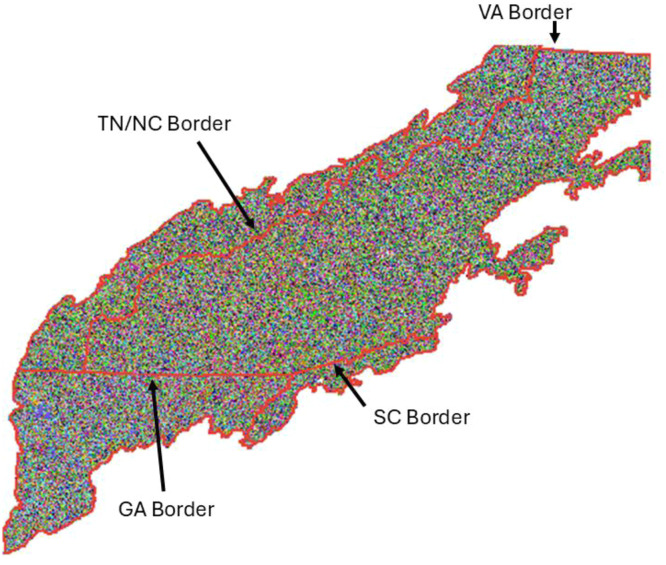
The “ricb_initial_communities.tif” for the SA (southern Appalachian) shapefile used in Fig. 1. State borders (red) were added for reference. The colors correspond to “MapCodes” that link with the “ricb_initial_communities.csv” lookup table to generate the appropriate species-age-biomass cohorts for that given MapCode (Table 4).

After species selection, RICB asks a user to input a time-step (Fig. 2C) which generates the age bins required for the species-age-biomass cohorts LANDIS-II requires from the dataframe. Once the species-age component of the planned cohorts are created, RICB then sums all the associated biomass values in a given species-age cohort by MapCode and weights the summed biomass value by pixel area to convert biomass to g/m^2^, the units required for LANDIS-II. The resulting file (Fig. 2D) with the mapcode-species-age-biomass cohorts is then outputted to a “.csv” file with an associated map linking the mapcodes. These outputs are respectively: ricb_initial_communities.csv (Table 5), and ricb_initial_communities.tif (Fig. 4).

Additionally, a list that relates the SpeciesName (Table 4) in the ricb_initial_communities.csv file to the species common name is provided to aid the user in parametrization of these species in the “parameterization_list.csv” file (Table 5). All files are found under the “landis_initial_files” folder. The user now has the required initial community inputs to run LANDIS-II for their relevant species.

**Table 4:** The first three “MapCodes” from the ricb_initial_communities.csv and the associated species-age-biomass cohorts that are used for the initial communities in LANDIS-II.

MapCode	SpeciesName	CohortAge	CohortBiomass	WoodBiomass	LeafBiomass
1	PITA	15	4519	4067	451
1	PITA	20	1553	1397	155
2	PITA	5	8	7	0
2	PITA	15	124	111	12
2	PRSE2	15	12	10	1
2	PRSE2	20	19	17	1
3	PIVI2	65	640	576	64
3	QUFA	40	310	279	31
3	QUFA	50	401	360	40

**Table 5:** The first four species in the “parameterization_list.csv”. “SPECIES_SYMBOL” matches “SpeciesName” in the ricb_initial_communities.csv file, while the “COMMON_NAME” is provided to aid in species parameterization without having to lookup what species belongs to what abbreviation.

SPECIES_SYMBOL	COMMON_NAME
ACRU	red maple
ACSA3	sugar maple
FAGR	American beech
HACA3	Carolina silverbell

## Method validation

**Fig. 5:**
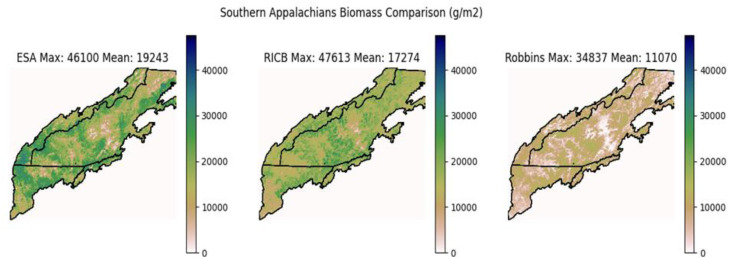
ESA vs RICB vs Robbins/Wilson Inital Biomass Comp.

We provide here an evaluation of our methodology by comparing to other published imputation methods. One of the largest continous areas in Region 8 that ran LANDIS-II is a portion of the Southern Appalachians [14] that was ∼8.5 million acres ranging from northern Georgia to the top of North Carolina (Fig. 4). For additional analysis, we accessed the European Space Agencys (ESA) imputation [27] of total aboveground biomass corresponging to this area. We then compared the total initial communitiy biomass values (Fig. 5) of RICB against these imputations. In general, the same patterns were shown but average biomass increased as a product became more recent. This is to be expected as the previous LANDIS-II imputation was based on Moderate Resolution Imaging Spectroradiometer (MODIS) imagery data from Wilson et al. [28] that used a nearest neighbor approach, TreeMap depends on Landsat data with its most recent release in 2022, and the ESA releases yearly (2024). The ESA product is also augmented with data from the Global Ecosystem Dynamics Invesitagion (GEDI), which placed a LiDAR instrument on the International Space Station (ISS). The directive of GEDI was to improve remote sensing biomass estimates in densly vegetaded ares by adding a vertical structural component not previously captured in other remote sensing techniques [29]. Therefore, as a product becomes more recent and incoporates newly available remote sensing data, we would expect to see densly vegetated areas become more pronounced. The fact that all three imputations display the same general trends in high/low biomass is encouraging, and the trend of seeing increasing initial biomass estimates with newly released remote sensing products expected. Only a qulatative anaylsis is provided as remote sensing does not directly measure AGB, it imputes it [29].

The previous LANDIS-II imputation [14] allowed for a further layer of evaluation as it also contains individual species information. Therefore, as total initial biomass differed, we compared the percentage of initial aboveground biomass predicted by the previous imputation and RICB (Table 6). When the top 10 species by %AGB on the landscape were considered, nine of the top 10 species were the same and these species accounted for 75–80 % of total initial AGB. In the previous method, sweet birch would replace black oak as a top 10 species corresponding to 3 % of total AGB. These rough cross comparisons support that RICB is comparable with other published methods.

**Table 6:** RICB vs Robbins et al. [14] top 10 species by percent aboveground biomass for the Southern Appalachians.

Top 10 Species by %AGB for RICB vs. Robbins et al. 2022		
Species	%AGB RICB	%AGB Robbins
chestnut oak	19	21
scarlet oak	10	7
white oak	9	8
red maple	9	11
yellow-poplar	7	12
northern red oak	7	9
black oak	5	1
sourwood	3	4
pignut hickory	3	3
eastern white pine	3	3

## Limitations

RICB as currently developed is limited to the eastern United States and only available on the Windows Operating System (OS). However, as mentioned in the “Biomass and Age Calculations” section, the “landis_tiles” created are for CONUS of each version of TreeMap. Meaning, if a user felt that age calculations produced from the site index curves of the eastern tree species sufficiently translated out west they would only need to slightly modify the “__RICBv1.py” script provided on the resource page to use RICB anywhere in the CONUS. RICB clips by USFS Region 8 or 9 for computational efficiency. Ignoring that clip and using the original TreeMap “.tif” file would enable expansion to CONUS. Our expertise are in eastern forests which is why the domain is currently limited.

Similarly, the “__RICBv1.py” script can be run on any non-Windows OS where Python is enabled. Typically, slight modifications are required when moving between operating systems. Additionally, PyInstaller is available on other operating systems such as MacOS and Linux, that many research labs use, it just is not a cross-compilier. Meaning, after the Python script was modified, if needed, for a different OS a user could run PyInstaller on that OS to create a compatible version of RICB for that OS. Here, we provided a GUI for the USFS regions we felt comfortable applying site index curves for age calculations to for an OS commonly used. All pre-processing code is available on the Rescource Availability website for potential expansion to CONUS and for use on different operating systems.

Finally, while RICB is useful for reducing the time it takes to populate LANDIS-II with initial communities a user must still currently possess the technical skills to run a LANDIS-II succession extension which limits its application to land management decisions. Ongoing work is focused on expanding RICB to incorporate some of the succession extensions available. Once complete, the goal is to rapidly reduce the time it takes a user to do a full LANDIS-II scenario run. RICB does have standalone utility while these upgrades are undertaken.

Our testers (see acknowedgments) found that shapefiles with corrupt geometeries need to be corrected befeore use with RICB.

## Ethics statements

No ethics statements to declare.

## CRediT author statement

**Steven A Flanagan**: Conceptualization, Software, Data Curation, Writing – Original Draft. **Zachary J. Robbins**: Methodology, Data Curation, Writing- Review & Editing. **Mac A. Callaham Jr**: Funding acquisition. **J. Kevin Hiers**: Conceptualization, Funding acquisition. **Brian R. Miranda**: Writing – Review & Editing. **Joseph J. O’Brien**: Funding aquisition. **Robert M. Scheller**: Methodology, Data Curation, Writing- Review & Editing, Resources. **E. Louise Loudermilk:** Conceptualization, Writing – Review & Editing, Supervision, Funding acquistion.

## Declaration of competing interest

The authors declare that they have no known competing financial interests or personal relationships that could have appeared to influence the work reported in this paper.

## Data Availability

All data and code posted on Zenodo. DIO provided in "Resource Availability" part of "Specifications table"

## Appendix Supplementary materials

Image, application 1
