# Supplementary material for: The Rapid Initial Community Builder (RICB) V1.1 for LANDIS-II
Source: MethodsX. 2025 Dec 13;16:103765. doi: 10.1016/j.mex.2025.103765 (PMC12775994; doi:10.1016/j.mex.2025.103765)
Supplement: Supplementary file 1 [file mmc1.docx]

**Supplementary material *and/or* additional information [OPTIONAL]**

| Region 9 Species Adjustment | |
| --- | --- |
| E_SPGRPCD | Common Name |
| 2 | Loblolly pine |
| 6 | balsam fir |
| 9 | northern white-cedar |
| 28 | black oak |
| 36 | white ash |
| 37 | quaken aspen |

**Table S1:** RICB as currently constructed calculated ages from Carmean’s equations based on the species for each Eastern Species Group Code (E_SPGRPCD) that was most prevalent in Region 8 of the USFS. This table provides the 6 E_SPGRPCD that would have a different dominate species in USFS Region 9 and what that species is as a place to start if a user were to feel latitudinal adjustments were required for their site.

| Code | Common Name | Coefficients | | | | |
| --- | --- | --- | --- | --- | --- | --- |
|  |  | b1 | b2 | b3 | b4 | b5 |
| 1 | longleaf pine | 1.421 | 0.9947 | -0.0269 | 1.1344 | -0.0109 |
| 2 | shortleaf pine | 1.7327 | 0.9998 | -0.0384 | 1.143 | -0.0004 |
| 3 | virginia pine | 1.2096 | 1.014 | -0.038 | 1.3247 | 0.0374 |
| 4 | eastern white pine | 1.966 | 1 | -0.024 | 1.8942 | 0 |
| 5 | jack pine | 2.0141 | 0.8989 | -0.0236 | 7.9649 | -0.5084 |
| 6 | red spruce | 1.3307 | 1.0442 | -0.0496 | 3.5829 | 0.0945 |
| 7 | eastern hemlock | 2.1493 | 0.9979 | -0.0175 | 1.4086 | -0.0008 |
| 8 | pondcypress | 1.3213 | 0.9995 | -0.0254 | 0.8549 | -0.0016 |
| 9 | eastern redcedar | 0.9276 | 1.0591 | -0.0424 | 0.3529 | 0.3114 |
| 23 | common pinyon | 0.9276 | 1.0591 | -0.0424 | 0.3529 | 0.3114 |
| 25 | white oak | 0.5605 | 1.3105 | -0.0145 | 0.1779 | 0.4323 |
| 26 | northern red oak | 0.4737 | 1.2905 | -0.0236 | 0.0979 | 0.6121 |
| 27 | chestnut oak | 1.9044 | 0.9752 | -0.0162 | 0.9262 | 0 |
| 28 | water oak | 1.3466 | 0.959 | -0.0574 | 8.9538 | -0.3454 |
| 29 | mockernut hickory | 1.8326 | 1.0015 | -0.0207 | 1.408 | -0.0005 |
| 30 | yellow birch | 2.2835 | 0.9794 | -0.0054 | 0.5819 | -0.0281 |
| 31 | sugar maple | 6.1308 | 0.6904 | -0.0195 | 10.1563 | -0.533 |
| 32 | red maple | 2.9435 | 0.9132 | -0.0141 | 1.658 | -0.1095 |
| 33 | American beech | 29.73 | 0.3631 | -0.0127 | 16.7616 | -0.6804 |
| 34 | sweetgum | 1.5932 | 1.0124 | -0.0122 | 0.6245 | 0.013 |
| 35 | blackgum | 1.5932 | 1.0124 | -0.0122 | 0.6245 | 0.013 |
| 36 | green ash | 1.6505 | 0.9096 | -0.0644 | 125.7045 | -0.8908 |
| 37 | eastern cottonwood | 1.3615 | 0.9813 | -0.0675 | 1.5494 | -0.0767 |
| 38 | American basswood | 4.7633 | 0.7576 | -0.0194 | 6.511 | -0.4156 |
| 39 | yellow-poplar | 1.2673 | 1 | -0.0331 | 1.1149 | 0.0001 |
| 40 | black walnut | 2.2349 | 0.842 | -0.0808 | 15.0884 | -0.6292 |
| 41 | black cherry | 7.1846 | 0.6781 | -0.0222 | 13.9186 | -0.5268 |
| 42 | black locust | 0.968 | 1.0301 | -0.0468 | 0.1639 | 0.4127 |
| 43 | sourwood | 7.1846 | 0.6781 | -0.0222 | 13.9186 | -0.5268 |
| 48 | honey mesquite | 0.968 | 1.0301 | -0.0468 | 0.1639 | 0.4127 |

**Table S2:** The site index coefficients chosen for each dominant species (Table 1) as determined by optimal fit.
